# Self-Care in Palliative Healthcare Professionals: A Qualitative Study

**DOI:** 10.3390/nursrep15050139

**Published:** 2025-04-25

**Authors:** Andrea Bovero, Maria Federica Spada, Alessandra Loreta Cito, Alexa Victoria Pidinchedda, Chiara Tosi, Sara Carletto

**Affiliations:** 1 Clinical Psychology Unit, AOU “Città della Salute e della Scienza” Hospital, Corso Bramante 88, 10126 Turin, Italy; m.federicaspada@gmail.com (M.F.S.); cito.alessandra@gmail.com (A.L.C.); alexapidinchedda@icloud.com (A.V.P.); chiara.tosi.88@icloud.com (C.T.); sara.carletto@unito.it (S.C.); 2 Department of Clinical and Biological Sciences, University of Turin, 10043 Turin, Italy

**Keywords:** self-care, healthcare professionals, palliative care, end-of-life care, focus groups

## Abstract

**Background/Objectives**: Self-care strategies enhance well-being and facilitate coping with emotional distress, particularly for palliative care professionals dealing with end-of-life issues. This study aimed to explore self-care practices among healthcare professionals working in palliative care settings by analyzing their perceptions and reflections. **Methods**: A total of 36 palliative care professionals participated in one of four focus group discussions on the topic of self-care. The discussions were transcribed and analyzed using qualitative content analysis. **Results**: Participants identified several self-care strategies as the most effective and the most frequently used, including lifestyle and emotional coping techniques. The meaning of self-care and its functions were delineated. Participants also identified factors that either facilitate or hinder self-care and discussed its impact on team well-being. Not all participants had received professional self-care training, and some struggled to identify their own strategies. However, participation in the focus group discussions was perceived as beneficial for improving work dynamics, group cohesion, communication, and freedom of expression. **Conclusions**: The positive feedback from the focus groups suggests that they are a valuable tool for fostering further discussions on self-care. The study recommends increasing the implementation of self-care strategies and professional training to enhance the well-being of palliative care professionals, their teams, and the patients they care for.

## 1. Introduction

Healthcare professionals working in oncological and palliative care settings are frequently exposed to distress, including moral distress [1,2] and burnout [3,4,5,6,7,8,9], due to daily contact with patients’ suffering, life-and-death clinical decisions, the end-of-life process, and death [9,10].

While empathy, defined as the ability to understand and resonate with another person’s emotions, is essential to provide compassionate care, it can become detrimental when it leads to excessive identification with others’ suffering. This can result in empathic distress and burnout, both associated with negative emotional states and adverse health outcomes [11].

Palliative care plays a crucial role in enhancing the quality of life of patients by addressing challenges commonly found in life-threatening illnesses, such as pain and physical, psychosocial, and spiritual suffering. By supporting both patients and their families, it aims to foster holistic well-being throughout the disease trajectory [12].

However, challenging working conditions (such as the management of difficult emotions, ethical conflicts, death anxiety, and heavy workloads) can negatively affect not only healthcare professionals’ well-being but also their work performance, as well as the quality of care and treatments [2,13,14,15]. The literature suggests that palliative care professionals can protect themselves and perform their work effectively if they are aware of the emotional and psychological risks associated with working in palliative care settings and implement self-care strategies [16].

Self-care involves intentional actions aimed at achieving, maintaining, and improving one’s health and well-being, as well as preventing and managing distress [17,18,19]. Self-care is a learned behavior through which individuals actively take care of themselves [17,19] in all aspects of their personal life, including physical, professional, relational, emotional, psychological, and spiritual well-being [18].

Caring for caregivers is essential to ensure the care of patients [14,20]; for this reason, implementing self-care is crucial not only for preserving the quality of life of palliative care professionals but also for ensuring high-quality end-of-life patient care [21]. Previous studies have shown a negative relationship between self-care and burnout [3,4,22], while positive relationships have been observed with work engagement [22] and compassion satisfaction [4,22]. According to Alkema et al. [4], compassion satisfaction and specific aspects of self-care related to emotional, spiritual, and personal–professional balance are positively correlated.

In the palliative care setting, healthcare professionals recognize self-care as essential for their well-being and job performance [16,23], and while its practice varies in frequency, it is generally considered a priority [23]. Self-care can be integrated into both personal and workplace settings [24] through physical, emotional, and social practices [23,25,26]. Cancer palliative care professionals who engage in self-care practices report significantly higher levels of psychological well-being, mastery, competence, positive relationships, and a sense of engagement and personal growth [27] compared to those who do not adopt such practices [28]. 

Research on self-care strategies in palliative care remains limited; this study aims to advance knowledge on this topic across different professional roles within this care setting. The chosen methodological approach is focus group interviewing, a qualitative method designed to collect in-depth data through focused discussion [29]. The focus group was used as a workshop to facilitate qualitative exploration and gather diverse perspectives. Specifically, through a qualitative analysis of perceptions and reflections concerning self-care, this study aims to examine how palliative care professionals define self-care, the most effective and commonly used strategies, their functions, the factors that support or hinder self-care, its impact on team well-being, the training received, and the perceived benefits of participating in this project.

## 2. Materials and Methods

The participants were recruited from the palliative care unit of one hospital and three hospices in Turin. The heads of the hospital and hospice departments were contacted, and the research project was shared with them. They then invited all team members to participate in the study. The inclusion criteria were working in palliative care settings and being able to understand and express oneself in Italian. All participants were reassured that their participation was voluntary, and that they were under no obligation to accept the invitation. Informed consent was obtained from all participants, and their privacy was guaranteed. 

The study was approved by the “Comitato Etico Aziendale A.O.U Città della Salute e della Scienza di Torino”: protocol number 0034403, procedure number CS2/1178, and was in accordance with the Declaration of Helsinki.

A focus group is a planned discussion with a small group of participants guided by a skilled moderator through a set of sequenced questions focused on a key topic [30]. The goal of a focus group is to gain participants’ insights on a specific topic [30].

Four focus group discussions were conducted to explore the topic of self-care, involving 36 out of the 45 professionals invited from the four teams at the hospital and the three hospices. Each team participated in one separate focus group session, with each lasting approximately one hour. The discussions took place at the participants’ respective workplaces, in person. Before starting the discussions, the focus group conductors, psychologists–psychotherapists experienced in palliative care (A.B., C.T.), provided an introduction to the theme of self-care.

According to a review of the previous literature [23,24,31], nine guiding questions were developed to conduct the discussions (Table 1). 

Sociodemographic data of participants were collected and analyzed through a descriptive analysis. 

Focus group discussions were recorded, transcribed verbatim and analyzed through a thematic content analysis to qualitatively identify and categorize the emerging themes inductively from the data [32]. The analysis was conducted by four researchers (A.L.C., A.V.P., C.T., M.F.S.), without the use of specific qualitative analysis software tools. The content analysis took three steps. First, each researcher independently categorized the emerging themes from the verbatim transcripts of discussions. Then, three of the researchers compared their individual categorizations for every theme that emerged. Lastly, all four researchers simultaneously compared their categorizations with those of the fourth researcher to determine the final themes. In this way, key themes emerging from the focus group discussions for each of the nine questions were identified. 

## 3. Results

### 3.1. Descriptive Analysis of the Sample

Out of a total sample of 45 palliative care professionals invited to participate in the study, 36 agreed to take part. Among those who did not participate, some declined the invitation and did not provide consent (N = 3), while others were unable to attend due to work shifts that conflicted with the focus group sessions (N = 6).

Each participant attended the focus group discussions in person with their own team from the hospital or the three hospices. The composition of each group was as follows:

- focus group 1: eleven participants;
- focus group 2: eleven participants;
- focus group 3: six participants;
- focus group 4: eight participants.

Among the participants, nine were male (25%) and twenty-seven were female (75%). The mean age was 47.08 ± 12.08 years, with a range from 22 to 67 years. The sociodemographic data of the sample are presented in Table 2.

### 3.2. Qualitative Content Analysis

For each of the nine questions presented following the order shown in Table 1, the answers are provided. The emerging themes from each question were identified and categorized through qualitative content analysis of all the transcripts of focus groups discussions (Table 3).

#### 3.2.1. First Question: “What Self-Care Strategies Do You Implement Most Frequently?”

Based on the participants’ responses during the focus groups, the following themes emerged:

- lifestyle strategies: taking care of physiological and physical aspects, such as maintaining a healthy lifestyle, ensuring restorative sleep and a balanced diet, taking care of oneself and one’s commitments, incorporating aromatherapy, and using essential oils;
- emotional coping strategies: physical isolation, finding moments of solitude to reconnect with oneself, maintaining a positive attitude, reflecting, crying, being able to disconnect from work at the end of the day;
- meaningful private life relationships: hanging out with loved ones, friends, relatives, and people who work in different professional settings and with whom you feel comfortable;
- taking care of others: volunteering, taking care of loved ones, patients, animals;
- psychological interventions: both individual (psychotherapy) and group and within the work context (supervision and psychodrama);
- discussion and sharing in teams: use of these strategies within the team;
- spiritual practices: meditation, yoga, prayer;
- physical activities: physical movement, walking, cycling;
- personal hobbies and passions: music, writing, reading and poetry, housework, cooking, handicrafts, shopping;
- contact with nature: trekking, taking care of plants.

#### 3.2.2. Second Question: “Which Ones Do You Consider Most Effective? Why?”

The following self-care strategies were identified as the most effective:

- lifestyle strategies: maintaining regular sleep and eating habits to re-center oneself, while keeping a separation between work and personal life;
- emotional coping strategies: being aware of one’s emotions, isolating oneself and being silent, finding time for oneself;
- meaningful private life relationships: spending time with friends and family;
- discussion and sharing in teams: discussion, sharing, and mutual support;
- spiritual practices: individual and community prayer;
- physical activities: physical movement, sports, walking, cycling to feel free, achieve physical well-being, focus on the present moment, and as a challenge. Dance Movement Therapy is useful as an activity carried out in the company of a group;
- personal hobbies and passions: taking trips and new experiences to slow down and stop, listening to music, reading, doing housework, cooking;
- contact with nature: admiring nature, landscapes, plants, and the proximity of pets with the serenity and tranquility they convey.

#### 3.2.3. Third Question: “What Does Taking Care of Yourself Mean to You?”

For the participants, taking care of themselves means the following:

- self-awareness and self-knowledge: listening to oneself, knowing oneself, being self-aware, turning attention inward, accepting oneself and one’s limits, and forgiving oneself;
- connecting with one’s emotions: having deep contact with one’s emotions and feelings and knowing how to listen to them;
- seeking work-life balance: avoiding the transfer of personal problems and negative emotions into the workplace, and vice versa;
- taking time for oneself: taking care of oneself by attending to the physical and spiritual aspects, having a healthy lifestyle, making good use of time, stopping and momentarily freeing oneself from worries, practicing personal hobbies and spending time with loved ones and friends.

#### 3.2.4. Fourth Question: “In Your Opinion, What Is the Function of Strategies You Implement?”

The self-care strategies implemented are aimed at the following:

- personal growth: inner and personal growth, finding oneself and one’s balance, and gaining deeper self-knowledge;
- working better: psychological serenity is reflected in the quality of work;
- improving relationships with oneself and others: establishing better connections with others, feeling a sense of belonging to a group, being in the right place, and feeling understood, non-judged, and supported by others;
- improving the quality of life: living better, feeling good, recalibrating priorities, achieving calm and tranquility, and relaxing. Self-care strategies have a liberating, regenerative, and nourishing function;
- managing and overcoming stress: controlling anxiety and stress, developing resilience, avoiding getting caught up in work, and centering oneself.

#### 3.2.5. Fifth Question: “What Are the Factors (In Your Personal and Professional Life) That Facilitate Your Self-Care Practices?”

The following themes emerged as factors that facilitate the implementation of self-care practices:

- individual factors: mental flexibility, reliance on oneself and one’s own strength, deriving gratification from one’s work;
- factors related to the work context: the organization facilitates the optimization of time at work;
- inter-individual factors related to private life: good social network that can motivate healthcare professionals;
- inter-individual factors of the team: support from the team, facing difficulties together, engaging in confrontation, and sharing.

#### 3.2.6. Sixth Question: “Conversely, What Are the Factors That Hinder Your Self-Care Practices?”

Factors that hinder self-care in palliative care professionals are as follows:

- organizational factors related to the work context: excessive workload and demand for performance for the time available, busy work under pressure and consequent difficulties in doing the own job well, and unforeseen events such as changes in work shifts;
- time-related factors: inability to disconnect from work, lack of time to devote to oneself, abuse and lack of respect for other people’s time;
- factors related to the team’s climate: an uncomfortable working environment, misunderstandings, lack of affinity between colleagues, and lack of support from them;
- private life factors: problems, worries, unforeseen events, lack of serenity and stress, related to factors external to professional life, and difficulty in committing to and self-imposing the act of taking care of oneself.

#### 3.2.7. Seventh Question: “Within Your Work Context, What Strategies, in Your Opinion, Can Improve Team Well-Being?”

Self-care strategies considered useful for improving the well-being of the team within the work context are as follows:

- personal aspects: personality and personal resources of healthcare professionals, open, welcoming, and non-judgmental attitude, the ability, possibility, and willingness to undertake a personal path of knowledge, self-awareness within the team context;
- relational aspects: quality relationships between healthcare professionals, care for the group, communication, sharing and comparison and mutual collaboration, support, availability, and recognition among colleagues;
- working environment: positive working environment, availability of the institution in times of difficulties, training of healthcare professionals, supervision, formalized meetings, clarity in work, roles and meetings, common goals and the same enthusiasm in achieving them, as well as the idea of spreading palliative care as a culture.

#### 3.2.8. Eighth Question: “Have You Ever Received Training in Self-Care Strategies During Your Professional Experience?”

Not all participants have received professional training related to self-care and, in general, a difficulty has been noticed in finding self-care strategies that are useful for themselves. In particular, only one hospice team (eight participants) received specific training on self-care. In addition, one healthcare professional attended a university course in nursing led by a psychologist that focused on the emotional and relational aspects experienced during the training.

#### 3.2.9. Ninth Question: “In Your Opinion, Was It Useful to Take Part in This Project and Discuss Self-Care? In Which Way? If Not, Why?”

All healthcare professionals found participation in this project and discussions about self-care useful. It was considered helpful for the following:

- improving work aspects and consolidating the group: discuss work aspects and problems, confront each other, make improvements, strengthen and bring the group closer together;
- sharing and listening: sharing one’s opinions with other participants and listening to those of others;
- learning to listen: listening to team members is important and useful for understanding their points of view and difficulties at work;
- reciprocal exchange: enriching the reciprocal exchange of opinions and self-care strategies implemented;
- freedom of expression: having the opportunity to freely express thoughts and opinions.

## 4. Discussion

This study examined the perceptions, reflections, and experiences of palliative care professionals regarding self-care, with a particular emphasis on those working in oncology and end-of-life care. Given the emotional and ethical complexity of cancer care, implementing effective self-care practices was essential for sustaining healthcare professionals’ well-being and preventing burnout. Consistent with previous research, our findings indicated that self-care is a multidimensional process that comprises physical, emotional, social, and spiritual components [23,26]. During focus group discussions, participants identified a range of self-care practices, including lifestyle modifications, emotional regulation strategies, meaningful interpersonal relationships, spiritual engagement, and team-based discussions. Our results also align with the key strategies identified by Mota Vargas et al. [16] for palliative care professionals to perform their duties effectively, such as recognizing one’s feelings, disconnecting from work during leisure time, and cultivating hobbies. These findings reinforced the notion that self-care is not a unidimensional concept but rather an integrative process that facilitates professional resilience and well-being, which was particularly crucial in the emotionally demanding field of oncology and palliative care [4,22].

A key finding of this study was the central role of self-awareness in effective self-care: our results indicate that self-care practices were most beneficial when professionals actively engaged in self-reflection and were able to recognize their own emotional and psychological needs. This aligns with previous research showing that self-awareness serves as a foundation for self-care, helping individuals identify stressors and develop adaptive coping mechanisms [19].

In oncology and palliative care settings, where professionals frequently encounter complex emotional challenges, self-awareness has become a crucial skill for maintaining empathy without experiencing empathic distress [11]. In particular, oncologists and palliative care nurses often experience high levels of moral distress due to the necessity of making difficult treatment decisions and witnessing patient suffering, further emphasizing the need for structured self-care strategies.

According to Mills et al. [24], our study showed that improving one’s wellness involves self-care strategies, encompassing both personal and professional aspects. Our study also emphasized the significant role of organizational support in either facilitating or hindering self-care practices. While individual responsibility for self-care was crucial, our findings revealed that institutional factors such as workload, work environment, and access to support systems greatly influenced the feasibility of self-care. These results align with existing literature emphasizing the importance of a supportive work environment in preventing burnout and enhancing professional quality of life, particularly in oncology and palliative settings [24], where healthcare professionals are continuously exposed to patient suffering and end-of-life decisions [7,16]. Moreover, the literature suggests that oncology healthcare professionals who engaged in regular supervision, peer support programs, or mindfulness-based interventions reported lower levels of burnout and higher levels of job satisfaction [26].

As highlighted in the literature [23], despite the recognized importance of self-care, many participants reported a lack of formal training on self-care strategies. This gap underscores the need for structured training programs that offer oncology and palliative care professionals the necessary tools to incorporate self-care into their daily practice. Previous studies suggest that interventions such as supervision, mindfulness training, and reflective practice groups were effective in fostering self-care behaviors and preventing compassion fatigue, which are particularly relevant for those working with cancer patients [33,34,35,36,37,38,39,40,41,42,43,44,45,46]. Given the unique emotional challenges of caring for cancer patients, integrating self-care training into oncology fellowship programs and continuing medical education initiatives could provide a sustainable approach to mitigate distress and foster long-term professional well-being.

The present study also shed light on the relational aspect of self-care, particularly within team dynamics. This finding is supported by the study of Mills et al. [24], which found similar results. Many participants of our study underline the importance of collegial support, shared experiences, and open communication as crucial elements of their self-care strategies. This finding was particularly relevant in oncology and palliative care settings, where interdisciplinary collaboration and emotional resilience were of prime importance [26]. Research in oncology nursing demonstrates that strong team cohesion and peer support significantly reduces stress and promotes job retention, highlighting the necessity of fostering workplace cultures that encourage collaborative self-care strategies [7].

This study has made significant contributions to the existing literature by allowing the exploration of self-care and how it is perceived and experienced within the context of oncology and palliative care. In terms of practical implications, this study highlights the critical importance of oncology and palliative care professionals’ understanding of self-care, emphasizing its clinical significance not only as a key preventive measure against distress, such as burnout and empathic distress, but also as a pivotal factor in enhancing their quality of life. By recognizing and identifying the factors that facilitate or hinder self-care, healthcare professionals could develop a deeper level of awareness of their strategies. The implementation of effective self-care strategies, as revealed in this study, could yield benefits for the quality of life and well-being of healthcare professionals working in the field of oncology and palliative care, thereby improving the quality of patient care.

Although this study provides valuable insights, it is not without limitations. The structured, survey-like format of the focus groups constrained the researchers’ ability to undertake a comprehensive exploration of the issues of self-care and their impact on healthcare provision. Moreover, prior to conducting the focus groups, their guide had not been validated by experts. The qualitative nature of the research, while rich in depth, limits generalizability. Future studies could employ mixed-method designs to quantitatively assess the impact of self-care interventions on burnout, work engagement, and patient care outcomes in oncology and palliative care teams.

Furthermore, our study relied on thematic content analysis conducted by multiple researchers. While this approach enhances validity, future research could benefit from employing qualitative software tools to ensure a more systematic coding process. Additionally, inter-rater reliability measures, such as Cohen’s kappa, could be incorporated to further strengthen the rigor of the analysis.

The present study had a modest number of participants. Expanding the sample size and including a more diverse range of palliative care settings, including home-based oncology and hospice care, could provide a broader perspective on self-care practices across different working conditions. Moreover, longitudinal studies examining changes in self-care behaviors over time and their relationship with professional well-being could offer deeper insights into the sustainability of self-care interventions. Given the high emotional toll of oncology care, future research should specifically investigate the effectiveness of different self-care interventions across medical oncologists, oncology nurses, and psychosocial oncology professionals.

## 5. Conclusions

This study contributed to a deeper understanding of self-care among oncology and palliative care professionals, emphasizing its critical role in resilience and job satisfaction. Self-care is a proactive and personalized approach to promoting health and well-being through a variety of strategies, in both personal and professional settings, to support the capacity to provide compassionate patient and family care. The findings of this research provide new insights to support palliative care practice and education. Institutional policies, professional training, and workplace initiatives regarding self-care are essential for reducing burnout and emotional distress, further improving professional well-being and the quality of patient care. Future research should explore innovative self-care interventions and assess their long-term impact on healthcare professionals’ quality of life and patient outcomes.

## Figures and Tables

**Table 1:** Focus group questions.

What self-care strategies do you implement most frequently?
Which ones do you consider most effective? Why?
What does taking care of yourself mean to you?
In your opinion, what is the function of strategies you implement?
What are the factors (in your personal and professional life) that facilitate your self-care practices?
Conversely, what are the factors that hinder your self-care practices?
Within your work context, what strategies, in your opinion, can improve team well-being?
Have you ever received training in self-care strategies during your professional experience?
In your opinion, was it useful to take part in this project and discuss self-care? In which way? If not, why?

**Table 2:** Descriptive analysis of the sample (N = 36).

	Categories	Frequency	Percentage (%)	Mean	SD
Sex	Male	9	25		
	Female	27	75		
Age	20–29	5	13.87	47.08	12.08
	30–39	4	11.11		
	40–49	10	27.78		
	50–59	11	30.56		
	60–69	6	16.67		
Workplace	Hospital	11	30.56		
	Hospice	25	69.44		
Profession	Nursing assistant	10	27.78		
	Nurse	13	36.11		
	Coordinator nurse	2	5.56		
	University nursing student	1	2.78		
	Physician	6	16.67		
	Psychologist	3	8.33		
	Spiritual assistant	1	2.78		
Working experience in palliative care (years)	0–2	14	38.89	8.98	8.02
	3–5	10	27.78		
	6–10	1	2.78		
	11–15	5	13.89		
	16–20	3	8.33		
	21–25	2	5.56		
	>26	1	2.78		

**Table 3:** Summary table of categorization of emerging themes for each of the questions and examples from transcriptions.

Questions	Emerging Themes	Examples
Most frequently implemented self-care strategies	Lifestyle strategies	“*[…] care of the organic and physical aspects.*”—P1
	Emotional coping strategies	“*[…] even just being with yourself in silence. […] it helps me to be alone with myself, in silence.*”—P2
	Meaningful private life relationships	“*[…] being with the people who make me feel good […]*”—P3
	Taking care of others	“*[…] I like to take care of the people I care about.*”—P4
	Psychological interventions (individual, group, work)	“*[…] the contribution of the psychotherapist with whom I have had a connection for years.*”—P5 “[Regarding the psychodrama supervision meetings] *[…] they represented […] a port where one landed and then departed […]*”—P6
	Discussion and sharing in teams	“*[…] be able to talk to your colleague about how you’ve felt. […] it happens here a lot […]*”—P7
	Spiritual practices	“*[…] prayer […] and the care of spirituality […] helps me to be more centered and balanced.*”—P8
	Physical activity	“*[…] moving my body […] to take care also of the emotional aspects, […] psyche and body are a unity*”—P1
	Personal hobbies and passions	“*I like to read poetry, […] cropping photos, […] doing handicrafts […] my little space that I take for myself.*”—P4
	Contact with nature	*“[…] going to the mountains […] to […] switch off and look at things from another point of view.*”—P9
Self-care strategies considered most effective	Lifestyle strategies	“*[…] regularity […] in both nutrition and sleep […]*”—P3
	Emotional coping strategies	“*[…] find a time of isolation in which I stay at home […] and I create an environment… a nest […] this thing […] isolates me from everything else and allows me to decant.*”—P10
	Meaningful private life relationships	“*[…] spending also time with family and friends.*”—P2
	Discussion and sharing in teams	“*[…] the beautiful thing […] that I observe in this group, is that there is a taking care of each other.*”—P11
	Spiritual practices	“*[…] what helps me the most […] is definitely prayer. […] without that I would have a much harder time finding a personal balance […]*”—P8
	Physical activities	“*[…] I cycle without noticing where I am, […] it helps me a lot. It frees me.*”—P12
	Personal hobbies and passions	“[Regarding traveling and new experiences] *[…] it helps me to detach myself and […] to distance myself […] to rework and see things from another perspective, but above all, to slow down and stop.*”—P3
	Contact with nature	“*[…] I try […] to go to my little garden, […] where I have planted all my plants […] I am content with that time that I take. […] in front of you there is the mountain, there is the meadow […] for the way I am, that is the best […]*”—P4
Meaning of self-care	Self-awareness and self-knowledge	“*[…] to enter deeper into oneself, to learn to know oneself deeply for what one is […]* [know] *just a little more […] of one’s soul […] of one’s heart, of one’s mind […]*”—P8
	Connecting with one’s emotions	“*Listen to my gut and make it say, because that’s the way it is, that it’s fine at that moment, regardless of what rationality would say […]*”—P10
	Seeking work–life balance	*“[…] we always try not to bring the work home and not to bring the home to the work […]*”—P13
	Taking time for oneself	“*In my opinion, caring is being able to stop, for a moment, to make a moment of emptiness and to get rid of all the thoughts that are circulating. […] be able to stop.*”—P14
Function of self-care strategies implemented	Personal growth	“*[…] as a term […] of knowledge, of personal deepening and certainly of growth […]*”—P8
	Working better	“*If you can take care of yourself, you can, of course, be calm and work better and better. That is, if we are mentally calm, surely we do our job better.*”—P15
	Improving relationship with oneself and others	“*It’s making me feel like I’m in the right place at the right time.*”—P16 “*[…] being able to relate better to people. It is not only a personal, intimate thing, which is certainly there, but this is a reflection and allows you to feel better with others as well.*”—P8
	Improving the quality of life	“[Self-care strategies] *[…] are liberating […] regenerative, nourishing […]*”—P5
	Managing and overcoming stress	“*[…] calm anxiety and stress […]*”—P4 “*[…] enhance your private life, not get absorbed in a very demanding job […]*”—P17
Factors facilitating self-care	Individual factors	*“The flexibility training you have in doing this job helps you a lot in your private life as well.*”—P17
	Factors related to the work context	“[The organization] *[…] allows you to […] move from one thing to another with a bit of logic without wasting time and managing to work with adequate time […]*”—P8
	Inter-individual factors related to private life	“*[…] also a good […] social network around. Something that pushes you to do […]*”—P18
	Inter-individual factors of the team	“*If there are difficulties, we face them together […]*”—P19
Factors hindering self-care	Organizational factors related to the work context	“*[…] the list of things to do quickly, the demand for performance […]*”—P6
	Time-related factors	“*[…] the whole day is so busy with work […] and sometimes you have little time to implement strategies […]*”—P1
	Factors related to the team’s climate	“*Not being able to understand each other, not being able to work together at that moment there.*”—P13
	Private life factors	“*[…] when I’m so […] polluted by external problems […] I’m drained, and I can’t take care of my patients.*”—P7 “*[…] it’s a much harder task than it sounds: the difficulty of self-obliging yourself to take care of yourself.*”—P20
Self-care strategies for the well-being of the team	Personal aspects	“*[…] the awareness of the person within a context and how this awareness can guide the person in his or her own self-care practices […]*”—P5 “*[…] an attitude of openness and, if possible, non-judgment.*”—P11
	Relational aspects	“*[…] we try to meet each other, […] there is a lot of attention among us operators.*”—P4 “*[…] make me available […] and know that this availability, in some way, is accepted and valued […]*”—P21
	Working environment	“*[…] clarity in work, […] in the roles, […] in the order of the meeting […]*”—P5 “*[…] supervision, meetings between operators, meetings.*”—P22 “*[…] having a common, shared goal and the same enthusiasm in wanting to achieve it […]*”—P10
Professional training on self-care	No	
	Yes	“*At the university there is a course called “Helping Relationship” […] we talk to the psychologist […] about what we experienced during the training, […] how to deal with certain situations.*”—P23
Perception of usefulness of participation in the project	Improving the work aspects and consolidating the group	“*[…] it also brings the operators very close […]*”—P24 “*This is the only way […] to confront and continue to cement the group […] there is always a need for small precautions […]*”—P9
	Sharing and listening	“*[…] sharing is very important […]*”—P7
	Learning to listen	“*It also makes us understand […] the difficulties that [the colleague] experiences in the workplace […]*”—P24
	Reciprocal exchange	“*[…] it also makes us understand what kind of strategies he* [regarding the other healthcare professionals] *uses and… strategies that maybe […] then we could also make our own.*”—P24
	Freedom of expression	“*[…] each of us was free to say what we wanted […]*”—P17

## Data Availability

All relevant data are included in the paper. The dataset is available from the corresponding author upon reasonable request.

## Public Involvement Statement

The participants were engaged through their involvement in the focus group discussions.

## Guidelines and Standards Statement

This manuscript was drafted in accordance with the SRQR (Standards for Reporting Qualitative Research: A Synthesis of Recommendations) for qualitative studies.
